# Dr. Jessie Shepard

**Published:** 1901-09

**Authors:** 


Dr. Jessie Shepard, the most prominent homeopathic woman physician in Buffalo, died Saturday, August 24, 1901, at the Homeopathic Hospital, after an operation for appendicitis. Dr. Shepard was taken ill Monday, August 19, and died two days after the operation was made. Dr. Shepard was a daughter of the late John D. Shepard, and a sister to Charles G. and Walter J. Shepard. She was born in Buffalo and obtained her early education in the public schools here. She attended the Homeopathic Hospital College in Cleveland and was graduated from the Boston University medical department.

Dr. Shepard was for one year resident surgeon in the Massachusetts Homeopathic Hospital. Afterwaid she practised in association with Dr. Joseph T. Cook, of Buffalo. A few years ago she visited Vienna, Austria, and spent nearly a year in the General Hospital there. She returned to Buffalo and resumed practice, so continuing until her last illness.

